# Supplementary material for: CDK inhibitors reduce cell proliferation and reverse hypoxia-induced metastasis of neuroblastoma tumours in a chick embryo model
Source: Sci Rep. 2019 Jun 24;9:9136. doi: 10.1038/s41598-019-45571-8 (PMC6591221; doi:10.1038/s41598-019-45571-8)
Supplement: Supplementary file 1 — Supplementary information [file 41598_2019_45571_MOESM1_ESM.pdf]

CDK inhibitors reduce cell proliferation and reverse hypoxia-induced metastasis of neuroblastoma tumours in a chick embryo model

Rasha R. Swadi[1], Keerthika Sampat[1 2], Anne Herrmann[1 2] Paul D. Losty[3 4], Violaine See[2], Diana J. Moss[1]*

Supplementary Figure 1

A

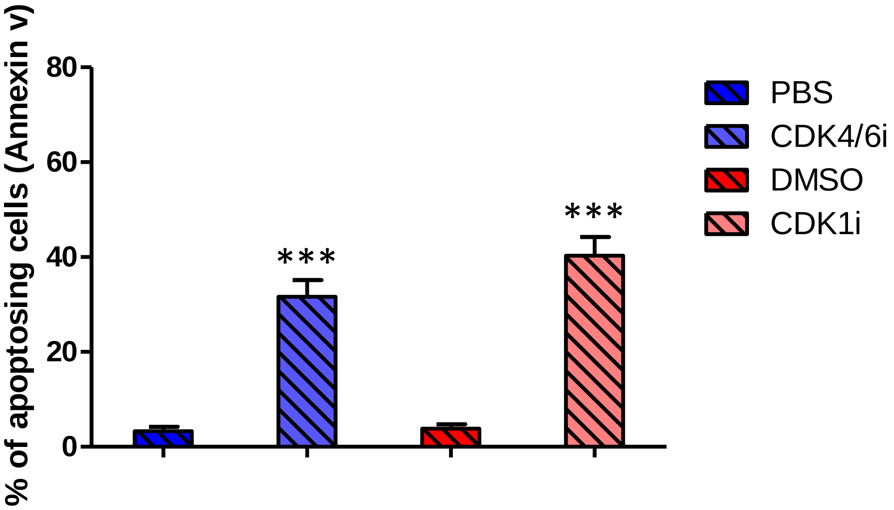

B

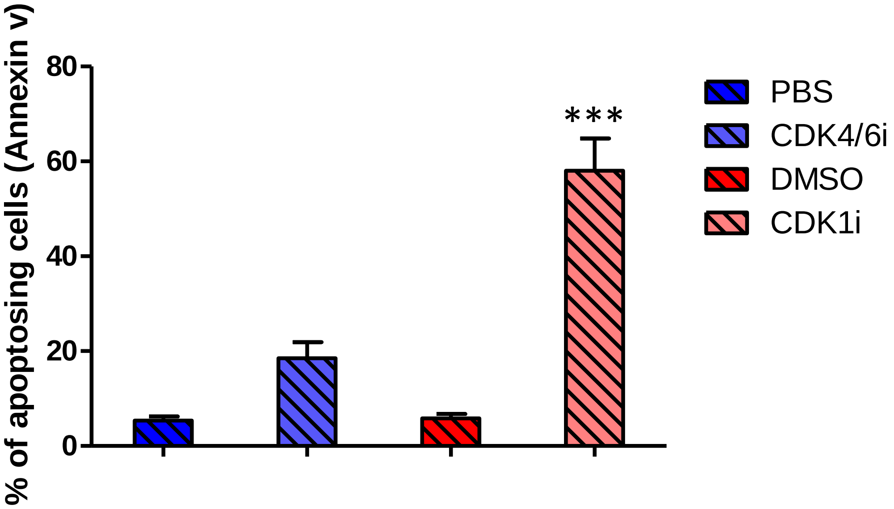

Supplementary Figure 2

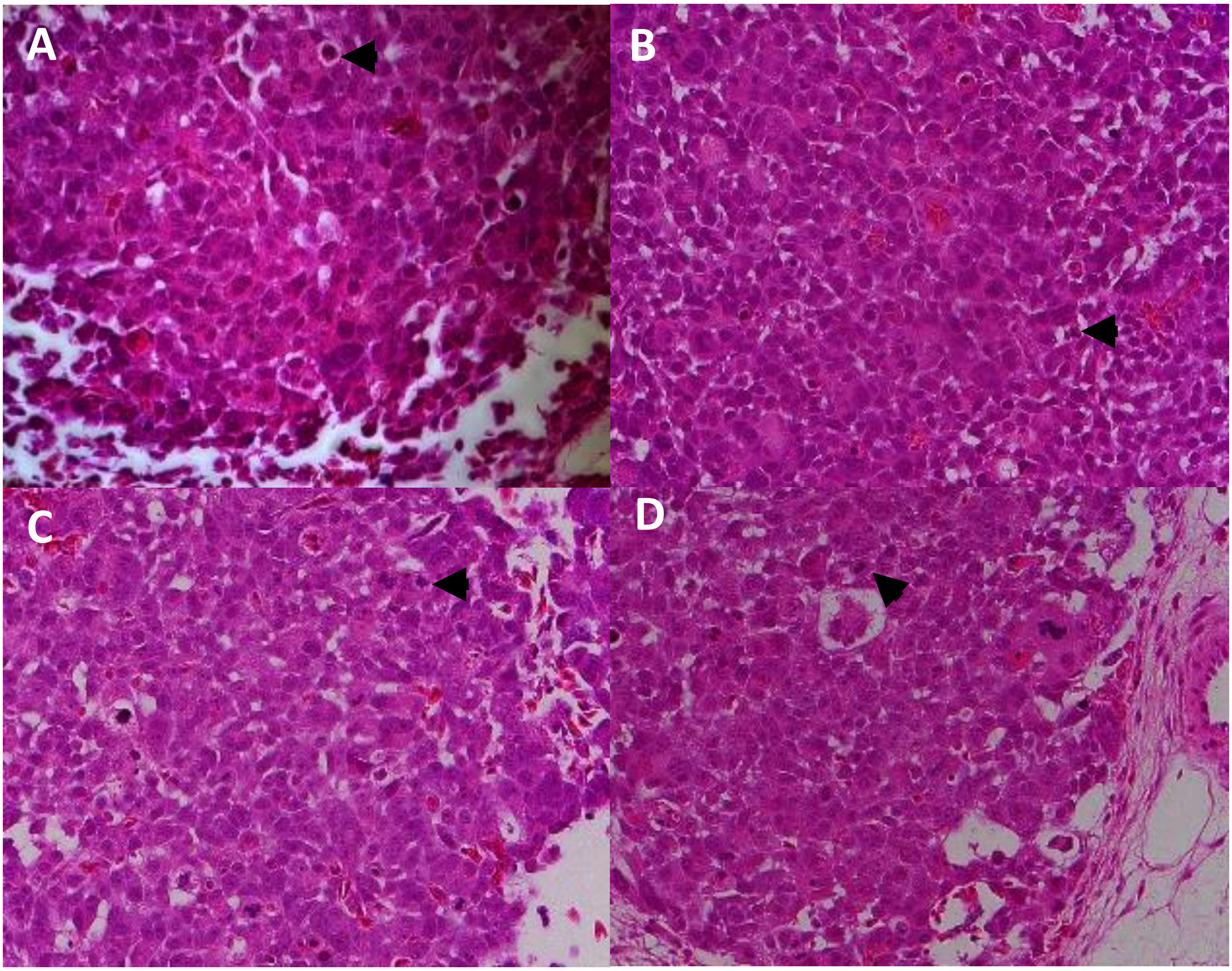

Supplementary Figure 3

A

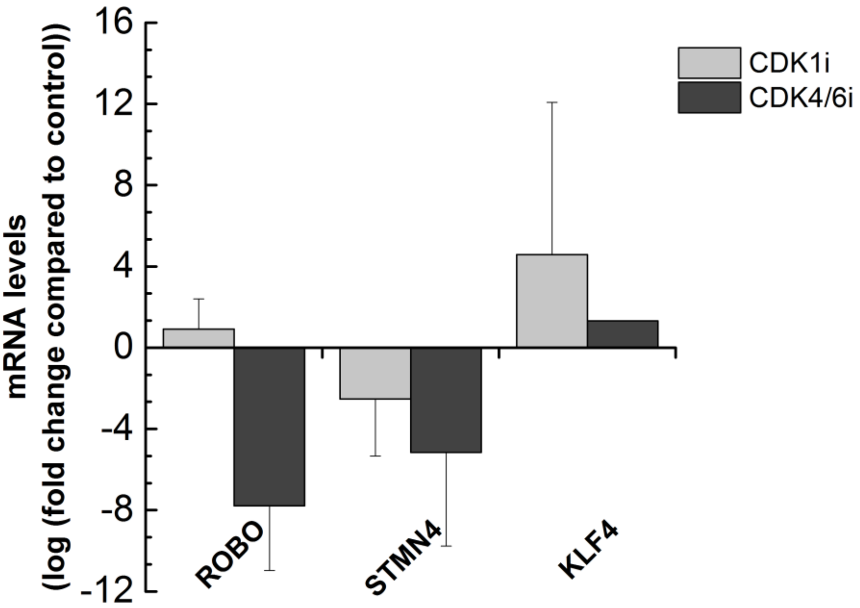

B

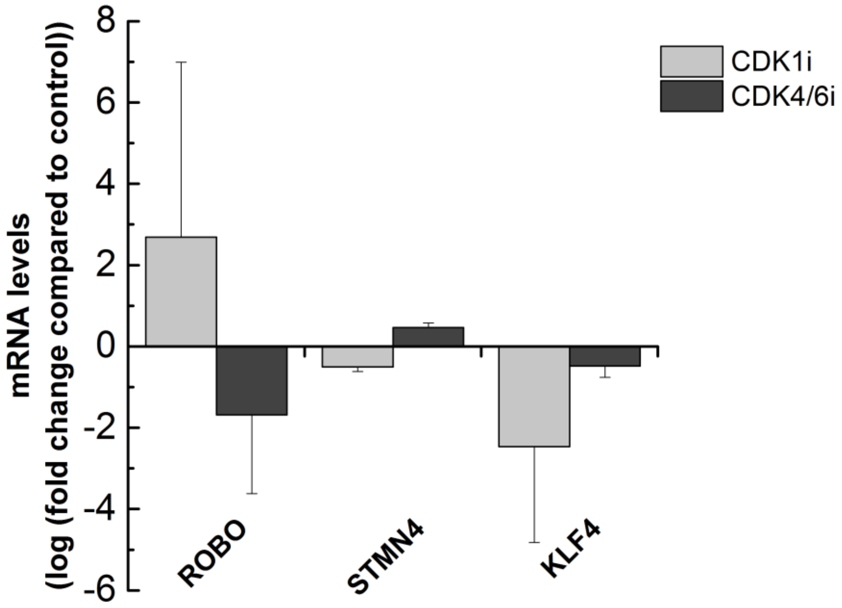

Supplementary figure 1:
Quantification of apoptosing cells in culture following treatment with either CDK4/6i or CDK1i. A. BE(2)C cells B. SK-N-AS cells.

Supplementary figure 2:
H&E stained sections of SK-N-AS treated neuroblastoma tumours. Tumours are from embryos treated with A) PBS – control for CDK4/6 inhibitor, B) DMSO – control for CDK1 inhibitor, C) CDK1 inhibitor D) CDK4/6 inhibitor. Black arrows depict apoptotic cells within the sample.

Supplementary figure 3:
Relative mRNA levels of **(A)** SK-N-AS or **(B)** BE(2)C tumours formed from cells grown in 21% O_2 for the target genes were determined by qPCR. Cells were implanted on the chick CAM at E7 and treated at E11 and E13 with either CDK1i or CDK4/6i or their respective controls (DMSO or PBS). At least three independent experiments (n = 3) were analysed for each condition and mRNA levels are displayed relative to GAPDH, UBC and HPRT1 and normalised to their respective control PBS (CDK4/6i) or DMSO (CDK1i). Each bar in the graph represents the normalised mean± SEM of at least three independent experiments.
